# Leyes de eutanasia en España y en el mundo: aspectos médicos

**DOI:** 10.1016/j.aprim.2021.102170

**Published:** 2021-11-16

**Authors:** Carmen Velasco Bernal, Jose Maria Trejo-Gabriel-Galan

**Affiliations:** Complejo Asistencial Universitario de Burgos, Burgos, España

**Keywords:** Eutanasia, Suicidio asistido, Ley, Legislación, Euthanasia, Assisted suicide, Law, Legislation

## Abstract

**Objetivo:**

Comparar la ley española de eutanasia y suicidio asistido con las que existen en otros países.

**Diseño:**

Revisión sistemática de la bibliografía.

**Fuentes de datos:**

Se buscaron en Medline/PubMed, EMBASE y Biblioteca Cochrane los estudios que contuvieran en su título o resumen los descriptores «eutanasia» o «suicidio asistido» y además «legislación» o «ley», entre 2002 y final de 2020.

Selección de estudios La búsqueda encontró 1.647 estudios y tras su cribado se valoraron 663, de los cuales 30 se incluyeron en la revisión. Se rechazaron estudios en los que solo constaran opiniones o no aportaran datos sobre la eutanasia/suicidio asistido en los países que los tienen regulados.

**Extracción de datos:**

Se registraron los criterios que regulan la aceptación o rechazo de una petición de eutanasia o suicidio asistido en España y en los demás países en que están despenalizados.

**Resultados:**

Las regulaciones de la eutanasia en el mundo pueden agruparse en tres: leyes que permiten la eutanasia y el suicidio asistido (Países Bajos, Bélgica, Canadá, algunos estados de Australia, Nueva Zelanda, España), en las que la ley solo permite el suicidio asistido (EE. UU.) y en las que se admite únicamente el suicidio asistido y en base a sentencias judiciales, sin legislación específica (Suiza, Alemania).

**Conclusiones:**

Aunque hay diferencias, las leyes a las que más se asemeja la ley española de eutanasia son las de Países Bajos y Bélgica, por lo que es previsible que la casuística de eutanasia y sus cifras en España se parezcan en el futuro a las de estos países.

## Abstract (en)

**Objective:**

To compare the Spanish law on euthanasia and assisted suicide with those that exist in other countries.

**Design:**

Systematic review of the bibliography.

**Data sources:**

Medline/PubMed, EMBASE and the Cochrane Library were searched for studies that contained in their title or abstract the descriptors «euthanasia» or «assisted suicide» and also «legislation» or «law», between 2002 and the end of 2020.

**Study selection:**

The search found 1647 studies and after screening 663 were assessed, of which 30 were included in the review. Studies that only contained opinions or did not provide data on euthanasia/assisted suicide in the countries that have them regulated were rejected.

**Data extraction:**

We registered the criteria that regulate the acceptance or rejection of a request for euthanasia or assisted suicide in Spain and in the other countries where they are decriminalized.

**Results:**

The euthanasia regulations in the world can be grouped into three groups: laws that allow euthanasia and assisted suicide (Netherlands, Belgium, some states of Australia, New Zealand, Spain), those in which the law only allows assisted suicide (USA) and those in which only assisted suicide is admitted and based on court decisions, without specific legislation (Switzerland, Germany).

**Conclusions:**

Although there are differences, the laws that the Spanish euthanasia law most closely resembles are those of the Netherlands and Belgium, so it is foreseeable that the casuistry of euthanasia and its figures in Spain will resemble that of those countries in the future.

## Introducción

La primera legislación despenalizadora de la muerte asistida por médicos, ya fuera eutanasia o suicidio asistido (E/SA), se aprobó en 2002 en Países Bajos y posteriormente se han sumado otros 6 países en el mundo (Bélgica, Luxemburgo, Colombia, Canadá, Nueva Zelanda y España) mientras que en Francia acaba de ser rechazada por el parlamento. El suicidio asistido (pero no la eutanasia) está regulado en Suiza, algunos estados de EE. UU. y Australia y en 2020 el Tribunal Constitucional alemán lo ha despenalizado. Los supuestos en los que la ley permite la E/SA tienen elementos comunes y diferenciales entre los distintos países. El objetivo de esta revisión es comparar entre ellos la legislación, las cifras y casuística de la E/SA y los factores sociosanitarios y de opinión.

## Material y métodos

Se realizó una revisión bibliográfica sistemática en Medline/PubMed, EMBASE y la Biblioteca Cochrane. No se buscó en bases de datos jurídicas por la orientación médica de la revisión. La búsqueda fue desde el año de la primera legislación reguladora de la eutanasia (enero de 2002) hasta diciembre de 2020, y limitada al español, inglés, francés y neerlandés, y a humanos. Se encontraron 1.208 publicaciones con la siguiente sintaxis en PubMed: ((“Eutanasia”[Title] OR “Euthanasia”[Title] OR “Euthanasie”[Title] OR “suicidio asistido”[All Fields] OR “assisted suicide”[Title] OR “suicide assiste”[All Fields]) AND (“Statistics”[All Fields] OR “Legislation”[All Fields]) AND (2002/01/01:2020/12/31[Date - Create]) AND(“english”[Language] OR “french”[Language] OR “spanish”[Language] OR “dutch”[Language])). Se encontraron 419 publicaciones con la siguiente sintaxis en EMBASE: (’law’:ab,ti AND’euthanasia’:ti,ab AND ([dutch]/lim OR [english]/lim OR [french]/lim OR [spanish]/lim) AND [humans]/lim AND [2002-2020]/py). Las publicaciones encontradas en la Cochrane Library estaban incluidas en las anteriores búsquedas.

## Resultados

**Figura 1:**
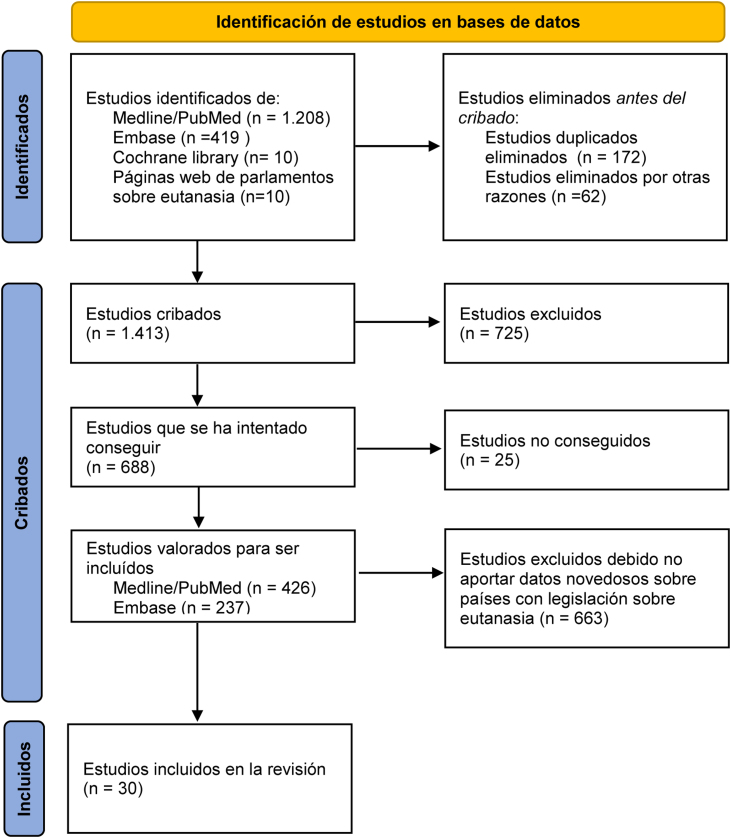
Diagrama de flujo tipo PRISMA^1^ para revisiones bibliográficas sistemáticas.

Siguiendo la metodología PRISMA^1^ (Diagrama de flujo del estudio, fig. 1), de las 1.647 publicaciones encontradas y tras excluir duplicados en diferentes bases bibliográficas se cribaron 1.413, de las que se excluyeron 725 por tratarse varias de ellas de opiniones del autor o ser respuestas a cartas al director sin aportar datos nuevos o por otros motivos; fueron valoradas para ser incluidas 688, de las que se pudieron conseguir 25. Tras el cribado quedaron 663 publicaciones y de ellas se eligieron las 30 que aportaron más datos sobre la E/SA en los países que los tienen regulados.

## Discusión

Se utilizará el término «eutanasia» tal como la define la ley española^2^: «aquella que se produce de manera activa y directa, de manera que las actuaciones por omisión que se designaban como eutanasia pasiva (no adopción de tratamientos tendentes a prolongar la vida y la interrupción de los ya instaurados conforme a la lex artis), o las que pudieran considerarse como eutanasia activa indirecta (utilización de fármacos o medios terapéuticos que alivian el sufrimiento físico o psíquico aunque aceleren la muerte del paciente —cuidados paliativos—) se han excluido del concepto bioético y juridicopenal de eutanasia». La ley española, a diferencia de las de otros países del mundo que lo han regulado, evita el término «suicidio asistido» y habla de «dos conductas eutanásicas diferentes, la eutanasia activa y aquella en la que es el propio paciente la persona que termina con su vida, para lo que precisa de la colaboración de un profesional sanitario».

### Legislación

Las leyes que regulan en 8 países del mundo la E/SA exigen por lo general que un enfermo adulto y competente para tomar decisiones tenga una enfermedad terminal o un sufrimiento intolerable que no haya mejorado con tratamientos previos, pida al médico la E/SA en dos ocasiones (de palabra y por escrito) separadas por varios días y casi siempre con un período de reflexión entre la última petición y la E/SA. El médico debe informar al paciente de su diagnóstico, pronóstico y de las alternativas de tratamiento, debe estar convencido de que el sufrimiento del paciente es intolerable, de que no hay un tratamiento eficaz, de que el enfermo es competente y actúa con libertad y por último debe comunicar a las autoridades la E/SA. Pero existen diferencias^3^ tanto en la legislación como en la práctica de la E/SA entre los diferentes países (Tabla 1, Tabla 2). Una primera diferencia entre países es que en algunos (EE. UU., Suiza) solo está autorizado el suicidio asistido, mientras que los demás, incluido España, permiten también la eutanasia. En EE. UU. el primer estado en legislar fue Oregón (1994) y le han seguido Washington (2008), Montana (2009), Vermont (2013), Colorado y la capital Washington DC (2016), California (2016), Hawái (2018), Nueva Jersey y Maine (2019). En Australia la E/SA están autorizados en el estado de Victoria y en el de Australia Oeste y en Nueva Zelanda la legislación entrará en vigor en noviembre de 2021.

**Tabla 1:** Principales aspectos de la legislación de los países que han regulado la eutanasia/suicidio asistido (E/SA)

País	Legalización	Características principales de la ley
Países Bajos	Primera ley en el mundo (abril de 2002) de eutanasia y suicidio asistido^4^; los médicos (sobre todo de cabecera y geriatras) tienen que informar de los casos a un Comité. Solo un médico ha sido procesado. Incluye enfermos mentales, dementes y menores	En estos 3 países del Benelux el enfermo debe pedir al médico, y más de una vez, la E/SA (el 95% pide eutanasia) debido a un sufrimiento físico o psicológico intolerable. El médico debe estar convencido de que no hay tratamiento eficaz, consultar a un colega médico independiente y comunicar a las autoridades las circunstancias de «cuidados apropiados» en que se ha realizado la E/SA
Bélgica	Ley de eutanasia^5^ septiembre 2002. Suicidio asistido no regulado pero tratado igual. Comisión a la que se informan casos	
Luxemburgo	Ley desde abril de 2009 de eutanasia y suicidio asistido^6^	
Suiza	Desde 1918 el artículo 115 del Código Penal despenaliza la asistencia al suicidio excepto si se hace «por motivos egoístas»^7^. Desde 1982, organizaciones no médicas asisten en el suicidio, también de extranjeros. No legalizada la eutanasia	Antes de prescribir la sustancia letal el médico debe informar al paciente solicitante de su pronóstico y alternativas y asegurarse de que es competente. Puede no estar presente cuando el paciente se la administra
Algunos estados de EE. UU. y Canadá	En EE. UU. está legalizado el suicidio asistido a enfermos terminales, pero no la eutanasia. Oregón^8^ fue el primero (1997). Eutanasia y suicidio asistido en Canadá desde 2016 a terminales y recientemente a enfermos no terminales tras 90 días de reflexión.	El paciente debe ser un adulto competente mentalmente, con una enfermedad terminal y el médico debe informarle de su diagnóstico, pronóstico, alternativas de cuidados paliativos, remitir a otro médico para que confirme el diagnóstico y que el paciente es competente, e informar a las autoridades
Colombia	La eutanasia legalizada no por ley sino por sentencia de la Corte Constitucional que ordenó al Ministerio de Salud elaborar un protocolo en adultos^9^ y niños^10^	Adulto en fase terminal sin enfermedad psiquiátrica cuya competencia valora un psiquiatra o psicólogo y un comité autoriza Niños a partir de los 6 años
Australia, Nueva Zelanda	Eutanasia y suicidio asistido despenalizados en estados de Victoria y Australia Oeste y lo estará en 2021 en Tasmania, Australia Sur y Nueva Zelanda	Adulto competente con sufrimiento intolerable que va a fallecer en 6 meses, hace petición verbal y escrita y es valorado por 2 médicos
España	Eutanasia y suicidio asistido aprobados por el Parlamento^2^ en marzo de 2021	Adulto competente con padecimiento grave, crónico o imposibilitante o enfermedad grave incurable con sufrimiento físico o mental insoportable que no se puede aliviar en condiciones que considere aceptables, que haga 2 peticiones escritas separadas por 15 días. Requiere informe de 2 médicos y una comisión

**Tabla 2:** Diferencia entre la muerte asistida entre grupos de países^2^, ^3^

	España	Benelux	EE. UU. y Canadá
% respecto a todas las muertes		4,6%	0,3%
Tipo de muerte asistida	Eutanasia y suicidio asistido	Eutanasia y suicidio asistido	EE. UU. solo suicidio asistido; Canadá eutanasia/suicidio asistido
Características de enfermedad	Sufrimiento intolerable	Sufrimiento intolerable	Sufrimiento intolerable y supervivencia < 8 meses
Enfermos mentales pueden solicitarla	No definido	Sí	No
Demencias pueden solicitarla	No	Sí	No
Solicitud por voluntades anticipadas	Sí	Sí	No
Menores pueden solicitarla	No	Sí	No
Plazo mínimo entre primera y última solicitud	15 días	30 días	Canadá 90 días en no terminales
Control por Comisión de garantía	Previo y posterior	Posterior	Posterior
Lugar donde fallecen pacientes		80% domicilio (Países Bajos)^15^	80% en centro de cuidados paliativos (Oregón)

Una segunda diferencia es el tipo de enfermos que pueden solicitar la E/SA: en EE. UU., Australia y Nueva Zelanda debe ser un adulto con enfermedad terminal (con supervivencia prevista menor de 6-8 meses), mientras que los demás países solo exigen un sufrimiento no tratable por otros medios. Una tercera diferencia es la eutanasia de menores (permitida por Países Bajos, Bélgica, Luxemburgo y Colombia, aunque raramente se pone en práctica) o de enfermos mentales (autorizada en Países Bajos, Bélgica, Luxemburgo y en Suiza cuando un psiquiatra certifica que la petición de suicidio no es consecuencia de la enfermedad mental). En España no se especifica que tener una enfermedad mental incumpla los supuestos de E/SA, lo que preocupa por la vulnerabilidad de estos enfermos^11^. En Suiza, aunque la prescripción de la sustancia la hace el médico, puede no ser este sino su familia o una organización privada de «derecho a morir» quien le asista durante el suicidio. En todos los países excepto Suiza y el estado de Montana, los médicos tienen obligación de notificar a las autoridades las muertes asistidas. En el Benelux, pero no en el resto de los países, se autoriza la eutanasia en demencias leves, y en las graves diferida a cuando se cumplan las condiciones expresadas previamente a la demencia en voluntades anticipadas. En Colombia, Alemania y Suiza, la despenalización de la E/SA en el primer caso, y solo del suicidio asistido en Alemania y Suiza, no proviene de una legislación del parlamento sino de sentencias del Tribunal Constitucional. La asociación médica alemana piensa que el suicidio asistido no es un tratamiento médico y opina que, como ya se hace en Suiza, debe ser personal no médico quien se encargue^12^.

La ley española indica que «... el contexto eutanásico, en el cual se acepta legalmente prestar ayuda para morir a otra persona, debe delimitarse con arreglo a determinadas condiciones que afectan a la situación física de la persona con el consiguiente sufrimiento físico o mental en que se encuentra, a las posibilidades de intervención para aliviar su sufrimiento…». Estas posibilidades se ven disminuidas en España por la menor disponibilidad de cuidados paliativos respecto a otros países que tienen regulada la eutanasia. Así, los equipos de cuidados paliativos por 100.000 habitantes que atienden al paciente en su domicilio/que le atienden en régimen de ingreso/y los que apoyan a los hospitalizados son^13^, respectivamente, España: 0,22/0,14/0,2; Bélgica: 0,25/0,47/1,02; Países Bajos: 0,11/0,41/0,36; Suiza: 0,41/0,63/0,38. En Bélgica la ley despenalizando la E/SA se acompañó de otra de acceso universal a los cuidados paliativos, y en España los sanitarios llevan demandando desde hace años una legislación específica de cuidados paliativos^14^.

### Cifras de intervenciones al final de la vida incluida la eutanasia/suicidio asistido y su evolución

En todos los países en los que se ha autorizado la E/SA las cifras han ido creciendo, siendo las más altas^15^ las de Países Bajos (4,2% de todas las muertes) y Bélgica^16^ (4,6%). En EE. UU. los suicidios asistidos fueron el 0,3% de las muertes totales en 2017, el triple que el primer año en que se aprobó la ley en el primer estado (Oregón), pero este es un porcentaje 10 veces menor que el del Benelux. Según una encuesta a médicos de familia de Bélgica, Países Bajos y Suiza, aproximadamente en la mitad de los pacientes que atendieron al final de la vida hicieron una intervención específica, la gran mayoría paliativas. Por orden de frecuencia fueron: tratamiento intensivo de los síntomas, retirada de tratamiento no paliativo, sedación terminal y lo menos frecuente fue la E/SA^16^, ^17^, ^18^ (tabla 3). Durante un período de 10-13 años aumentaron todas las intervenciones, incluida la eutanasia. En los países en que tanto eutanasia como suicidio asistido están despenalizados (Bélgica, Países Bajos), la eutanasia es 10 veces más frecuente que el suicidio asistido^17^.

**Tabla 3:** Evolución de las intervenciones al final de la vida (%)

	Flandes (Bélgica)		Países Bajos		Suiza germanohablante	
	2001	2013	2001	2010	2001	2013
Cualquier intervención al final de la vida	38,4	47,8	43,8	57,8	52	58,7
Tratamiento intensivo de síntomas	22	24,2	20,1	36,4	22	21
Retirada de tratamiento no paliativo	14,6	17,2	20,2	18,2	28	35
Sedación profunda hasta fallecimiento	8,2	12,3	5,6	12,3	4,7	17,5
Eutanasia	0,3	4,6	2,6	2,8	0,2	0,3
Suicidio asistido	0,01	0,05	0,2	0,1	0,3	1
Aceleración de fallecimiento sin petición expresa del paciente	1,5	1,7	0,7	0,2	0,5	0,8

No todas las E/SA solicitadas son aceptadas ni llevadas a cabo y Países Bajos informa a sus ciudadanos que la E/SA no es un derecho exigible. En una encuesta en Bélgica a 3.022 médicos generales, el 39% de ellos que había recibido solicitudes de E/SA de sus pacientes habían aceptado el 48% de las mismas, rechazado el 5%, el 10% de los pacientes había retirado la solicitud y el 23% había fallecido antes de que se llevara a cabo^19^, ^20^. En otra encuesta a médicos hubo errores entre el 20 y 30% cuando se pidió diferenciar en casos concretos entre eutanasia y tratamiento intensivo del dolor y qué casos debían ser comunicados a las autoridades como eutanasia^21^, ^22^.

### Casuística en que se aplica la eutanasia/suicidio asistido

En todos los países en los que la E/SA están regulados, aproximadamente la mitad de los casos se producen entre los 60 y los 85 años. Algo más de la mitad son varones excepto en Suiza y en el 2018 en Bélgica^3^ donde predominaron las mujeres. También de forma general el cáncer es la causa de más de la mitad de las solicitudes de E/SA y a continuación vienen las enfermedades neurológicas^15^, ^23^, y entre estas últimas la más frecuente es la demencia. Las E/SA por demencia se han multiplicado por 6 en Países Bajos entre 2010 y 2018, mientras que por el resto de las causas se han duplicado. La demencia interfiere con la capacidad de decisión, por lo que no entra dentro de los supuestos de la ley española que establece que no será posible la eutanasia cuando «… el médico responsable certifique que el paciente no se encuentra en el pleno uso de sus facultades…»^2^. La ley permite que el paciente establezca por voluntades anticipadas que se lleve a cabo la eutanasia si desarrolla un cierto grado de demencia, aunque entonces no sea ya competente, pero determinar el momento de la eutanasia porque ha alcanzado ese grado de empeoramiento y corroborar que existe un «sufrimiento insoportable» es difícil ante un enfermo al que no se le puede ya preguntar eso, y ni siquiera si ha cambiado de opinión. Puede no haber acuerdo sobre ello entre el representante y el médico. Estos problemas se darán en España, como ya se dan en Bélgica y Países Bajos, donde son causa de rechazo de muchas peticiones de eutanasia en demencias avanzadas en base a voluntades anticipadas^24^. Una eutanasia en estas condiciones es la única por la que un médico ha sido procesado en Países Bajos^25^. Otro problema de cualquier solicitud de E/SA es determinar cuándo es el «sufrimiento físico o mental insoportable que no se puede aliviar en condiciones que (el paciente) considere aceptables», como establece la ley española. En Países Bajos suele haber bastante coincidencia entre médicos respecto a cuándo el dolor físico es insoportable, pero es mucho menor cuando el sufrimiento no se debe a problemas físicos sino a la pérdida de la funcionalidad de la persona^26^.

### Factores sociosanitario y de opinión

En Bélgica, y con la misma ley, hay diferentes actitudes y prácticas de E/SA en el norte de habla flamenca y en el sur de habla francesa, lo que muestra la gran influencia sociocultural en la E/SA^22^. Dentro de Europa, en países como Gran Bretaña, Alemania e Italia, el apoyo de la opinión pública a la E/SA ha aumentado entre 1999 y 2008, en países del centro y este de Europa ha disminuido y en otros hay un rechazo mayoritario^12^, ^27^. En los países en los que la E/SA están aprobados hay más apoyo que rechazo a la misma entre la opinión pública, a veces por estrecho margen. En ellos, los sanitarios son en general menos favorables a esta forma de terminación de la vida que la población general^28^ y ello en proporción a su responsabilidad en llevarla a cabo: las enfermeras apoyan menos la E/SA que la población general, los médicos menos que las enfermeras y los médicos que cuidan enfermos susceptibles de pedir la E/SA (oncólogos, geriatras) menos que la generalidad de los médicos^29^, ^30^.

Una limitación de esta revisión es mostrar solo el aspecto clínico y no el jurídico-legal de la legislación sobre la E/SA, no recopilar iniciativas legales que no han salido adelante y no haber pretendido responder a una única pregunta estructurada, como es propio de las revisiones sistemáticas. Su fortaleza estriba en mostrar la legislación española dentro del panorama mundial.

## Conclusiones

En el mundo, la legislación despenalizadora de la E/SA solo existe en algunos países de «cultura occidental»: Canadá, algunos estados de EE. UU. y Australia, Colombia y algunos países de Europa. La legislación española recientemente aprobada se parece a la de Países Bajos y Bélgica, los países con mayores porcentajes de E/SA. En EE. UU. solo está autorizado el suicidio asistido.

El porcentaje de E/SA está aumentando en todos los países en que han sido legalizados.

**Lo conocido sobre el tema**

- Hay tres tipos de regulaciones de la eutanasia/suicidio asistido en el mundo: la ley permite la eutanasia y el suicidio asistido (Países Bajos, Bélgica, Canadá, algunos estados de Australia, Nueva Zelanda y España), la ley solo admite el suicidio asistido (EE. UU.), y no hay ley específica y solo se admite el suicidio asistido y en base a sentencias judiciales (Suiza, Alemania).
- En todos los países en que se ha aprobado la eutanasia, las cifras han aumentado con el paso de los años, siendo en 2019 el 4,2 y 2,4% de todas las muertes en Países Bajos y Bélgica, respectivamente.
- En Países Bajos la mayoría de las eutanasias las practican médicos de atención primaria en el domicilio del paciente, mientras que en EE. UU. el entorno más frecuente es un centro de cuidados paliativos.

**Qué aporta este estudio**

- Se compara la ley española de eutanasia con la de otros países.
- Las leyes de eutanasia de Países Bajos y Bélgica son las más parecidas a la española, por lo que es previsible que la casuística de eutanasia y sus cifras en España se parezcan en el futuro a las de estos países.

## Financiación

Los autores agradecen a la «Fundación Burgos por la investigación de la salud», sita en el Hospital Universitario de Burgos, por la financiación de los gastos de publicación Open Access.

## Conflicto de intereses

Los autores declaran que no tienen intereses económicos o relaciones personales que puedan haber influido en el contenido de este trabajo.
